# The Global Influenza Hospital Surveillance Network (GIHSN): a new platform to describe the epidemiology of severe influenza

**DOI:** 10.1111/irv.12335

**Published:** 2015-10-13

**Authors:** Joan Puig-Barberà, Anita Tormos, Svetlana Trushakova, Anna Sominina, Maria Pisareva, Meral A Ciblak, Selim Badur, Hongjie Yu, Benjamin J Cowling, Elena Burtseva, on behalf of the GIHSN Group

**Affiliations:** a Fundación para el Fomento de la Investigación Sanitaria y Biomédica de la Comunidad Valenciana (FISABIO)Valencia, Spain; b D.I. Ivanovsky Institute of VirologyMoscow, Russian Federation; c Research Institute of InfluenzaSt. Petersburg, Russian Federation; d National Influenza Reference Laboratory Capa-IstanbulIstanbul, Turkey; e Key Laboratory of Surveillance and Early-warning on Infectious Disease, Division of Infectious Disease, Chinese Center for Disease Control and PreventionBeijing, China; f Li Ka Shing Faculty of Medicine, School of Public Health, The University of Hong KongHong Kong, China

**Keywords:** Hospital, influenza epidemiology, surveillance network

## Abstract

**Background:**

Influenza is a global public health problem. However, severe influenza only recently has been addressed in routine surveillance.

**Objectives:**

The Global Influenza Hospital Surveillance Network (GIHSN) was established to study the epidemiology of severe influenza in consecutive seasons in different countries. Our objective is to describe the GIHSN approach and methods.

**Methods:**

The GIHSN uses prospective active surveillance to identify consecutive influenza admissions in permanent residents of well-defined geographic areas in sites around the world. A core common protocol is followed. After consent, data are collected on patient characteristics and clinical outcomes, respiratory swabs are obtained, and the presence of influenza virus and subtype or lineage is ascertained by polymerase chain reaction. Data are collated and analyzed at the GIHSN coordination center.

**Results:**

The GIHSN has run its activities for two consecutive influenza seasons, 2012–2013 and 2013–2014, and hospitals in Brazil, China, France, Russian Federation, Turkey, and Spain have been involved in one or both seasons. Consistency on the application of the protocol and heterogeneity for the first season have been addressed in two previous publications. During both seasons, 19 677 eligible admissions were recorded; 11 843 (60%) were included and tested, and 2713 (23%) were positive for influenza: 991 (37%) A(H1N1); 807 (30%) A(H3N2); 583 (21%) B/Yamagata; 56 (2%) B/Victoria and 151 (6%) influenza A; and 125 (5%) influenza B were not characterized.

**Conclusions:**

The GIHSN is a platform that provides information on severe influenza worldwide, applying a common core protocol and a consistent case definition.

## Introduction

Influenza infection results in significant morbidity and mortality. Influenza is believed to infect 10–20% of the population annually; 5–10% of adults and 20–30% of children will show some clinical manifestation and resulting in about 3–5 million cases of severe illness, and about 250 000–500 000 deaths.^1^–^4^ Influenza illness can result in hospitalization and death mainly among high-risk groups, but also in a substantial proportion of previously healthy subjects.^5^

Groups at particular risk of severe influenza include pregnant women, children aged <5 years, the elderly, and individuals with underlying health conditions such as HIV/AIDS, asthma, obesity, and chronic heart or lung diseases.^6^ Less is known about the impact of severe influenza in previously healthy subjects. Severe disease due to influenza only recently has been addressed in routine surveillance systems.^7^ The level of evidence to support risk factors for influenza-related complications is limited or absent, and some well-accepted risk factors, including pregnancy, are discussed.^8^ Prospective hospital-based studies with laboratory-confirmed endpoints are required to better assess the contribution of influenza to severe morbidity.^9^

Historically, influenza surveillance has focused on virological monitoring and collection of specimens to support vaccine strain selection. In recent years, especially after the 2009 pandemic season, influenza surveillance has been expanded as recommended by the World Health Organization (WHO) to include more epidemiological information to complement the virological data.^10^

Various existing networks monitor hospitalizations associated with laboratory-confirmed influenza. Some focus on the description of influenza circulation at a country level, such as Southern Hemisphere Influenza Vaccine Effectiveness Research and Surveillance (SHIVERS),^7^ sentinel hospitals in Australia,^11^ and the Influenza Hospitalization Network (FluSurv-NET) and the New Vaccine Surveillance Network (NVSN) in the United States,^12^ whereas others have a broader focus such as the Red para la Evaluación de la Efectividad de la Vacuna en Latino América y el Caribe – influenza (REVELAC-i) in Latin America,^13^ InHove in Europe,^14^ and more globally, International Network for Strategic Initiatives in Global HIV Trials (INSIGHT).^15^ Some networks seek to capture information generated as part of routine patient care and rely on a retrospective case-ascertainment approach; others perform active surveillance, applying different sampling strategies to enroll hospitalized patients in whom respiratory specimens are obtained independently of clinician ordering. Differences in the sources of information, case ascertainment, definitions of criteria for inclusion, and geographic span have an impact on the magnitude of disease burden estimates and of the relatedness of various risk factors with influenza.^16^ This makes it difficult to compare the influenza burden between countries and provide a global estimate of disease burden.^12^

Understanding the population burden of influenza, and the risk factors for influenza virus infection and influenza-associated severe disease, requires adequately powered studies that collect epidemiological and clinical data from laboratory-confirmed cases during various seasons, and using a consistent approach. The Global Influenza Hospital Surveillance Network (GIHSN), using a standardized protocol based on a prospective and common approach to case selection for testing and data collection, may fill this gap.

The GIHSN is a public–private partnership between Sanofi Pasteur, Lyon, France; Fundación para el Fomento de la Investigación Sanitaria y Biomédica de la Comunidad Valenciana (FISABIO), Valencia, Spain; Fondation Mérieux, Lyon, France; D.I. Ivanovsky Institute of Virology, Moscow, Russian Federation; Research Institute of Influenza, St. Petersburg, Russian Federation; National Influenza Reference Laboratory Capa-Istanbul, Istanbul, Turkey; Key Laboratory of Surveillance and Early-warning on Infectious Disease, Division of Infectious Disease, Chinese Center for Disease Control and Prevention, Beijing, China; and Li Ka Shing Faculty of Medicine, School of Public Health, The University of Hong Kong, Hong Kong, China. All of these institutions act as coordinating sites. Each coordinating site supervises a local network of hospitals in its country or geographic region and follows the GIHSN core reference protocol; FISABIO acts as the GIHSN coordinating center.

The main goals of the GIHSN are to describe circulating virus types and subtypes, and their relationship to global and regional patterns of severe disease; develop an understanding of the relationship of virus strains to severe disease, determine influenza-related burden of severe disease; and estimate how much severe disease is prevented by influenza vaccination.^5^,^17^

Here, we describe in detail the GIHSN approach and methods to attain these goals.

## Methods

The GIHSN runs a multicenter, prospective, active-surveillance, hospital-based influenza epidemiological observational study to collect epidemiological and virological data for the Northern and Southern hemispheres over several consecutive seasons. In each country or region, the study is run by a coordinating site. Coordinating sites are public health or academic institutions affiliated with national health authorities. Each coordinating site supervises a local network of hospitals in its country or geographic region and follows the GIHSN core reference protocol.

### Study population and study period

**Figure 1:**
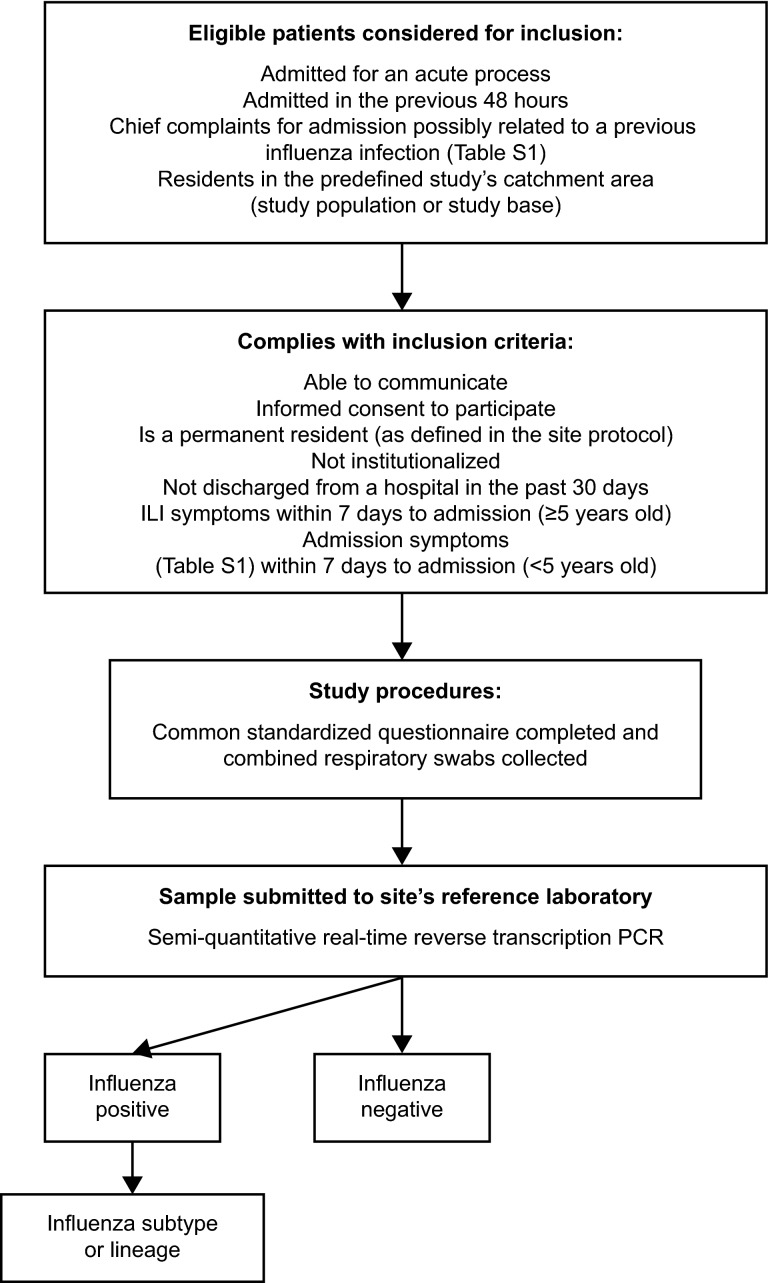
The GIHSN study flow diagram. PCR, polymerase chain reaction.

Each site defines a geographic or administrative catchment area from which eligible admissions in permanent residents (defined as residency of ≥6 months) are ascertained (Figure1). Depending on the site, this geographic catchment area may be a province, city, district, or neighborhood. For each site, this defines the study population or study base.^18^ Each site also predefines the criteria for the beginning of patient enrollment according to the site experience of the influenza epidemic.

### Study subjects

We consider for inclusion all consecutive admissions of non-institutionalized patients who are permanent residents in each site's catchment area and who are hospitalized because of a set of predefined chief complaints (Table S1).^19^,^20^ The patients must have arrived at hospital <48 hours before enrollment and must not have been discharged from a hospital within 30 days of the current admission (Figure1).

Once deemed eligible, for patients ≥5 years of age, we require the presence of at least one of four systemic symptoms (fever or feverishness, headache, myalgia, or malaise) and at least one of three respiratory symptoms (cough, sore throat, or shortness of breath), following the European Centre for Disease Prevention and Control clinical case definition of influenza-like illness (ILI),^21^ and being admitted within 7 days of the onset of ILI symptoms. Children <5 years of age are included if admitted to hospital within 7 days of the appearance of clinical conditions potentially associated with influenza (Table S1).

The list of chief complaints enumerated in Table S1 may be restricted to acute respiratory infection complaints when only acute respiratory infectious disease wards are participating. The time since arrival to hospital can be extended to 72 hours in subtropical regions where intermediate short stay filtering hospitals are part of the healthcare delivery process.

Study subjects are identified by healthcare professionals trained to follow the study protocol, who daily review hospital enrollment lists, electronic records, or other lists of admission diagnoses or symptoms.

The GIHSN study is to be approved by the local research ethics committees for each participating coordination site. Patients are included and respiratory swabs obtained after written or oral informed consent, depending on local ethics committees’ requirements.

### Data collection

A common standardized questionnaire is completed by face-to-face interview and by clinical records searching. Demographic, clinical, and epidemiological information is collected, including anthropometric measures, dates of symptom onset, hospitalization and swabbing, antiviral treatment received and its duration, intensive care unit (ICU) admission, death during hospitalization, main hospital admission and discharge diagnostics, presence of chronic diseases, pregnancy status, number of hospital admissions in the past 12 months, number of general practitioner consultations in the previous 3 months, and smoking habits. Social class is assigned according to occupation.^22^ Functional status before ILI onset is ascertained in patients ≥65 years of age using the Barthel index.^23^ We additionally collected information from the 2014–2015 season in the Northern Hemisphere and the 2015 influenza season in the Southern Hemisphere on mechanical ventilation and extracorporeal membrane oxygenation.

### Vaccination status

Influenza vaccination for the previous two seasons and for the current season is ascertained from registries, vaccination cards, physicians providing care, or by recall from patients or their families. Recall is considered as proof of vaccination. Subjects are considered vaccinated if there is a record or recall of at least one dose of the current season's seasonal influenza vaccine ≥14 days before the onset of illness. Those in whom the period between vaccination and onset of illness is <14 days are considered as unvaccinated.

### Sample collection

Combined respiratory swabs are obtained in all included subjects. A nasopharyngeal swab is obtained from all patients; in addition, a pharyngeal swab is collected for patients ≥14 years of age, and a nasal sample is taken for children <14 years old. Both swabs are collected into the same test tube and stored at least at −20°C at the study site or sent directly to the coordinating site's reference laboratory for testing.

### Confirmation of influenza infection

Laboratory confirmation is undertaken using real-time polymerase chain reaction (RT-PCR) assays to detect influenza A (subtypes H3 and H1) and influenza B (Yamagata and Victoria lineages) viruses in clinical specimens. The specimens are tested for influenza in the coordinating site's reference laboratories according to routine testing procedures following the WHO RT-PCR protocol.^5^

### Data management

Local coordination centers share anonymized data with the network coordinating center (FISABIO, Valencia, Spain). The coordination center checks for missing, inconsistent, or incorrect data, and queries are resolved by the investigators at each of the study sites. Missing data are not replaced for the statistical analyses. Those with missing laboratory results, date of illness onset, or vaccination data are excluded.

### Statistical analysis

#### Descriptive analysis

The primary outcome measure is hospital admission with laboratory-confirmed influenza. Secondary outcomes are hospital admissions with laboratory-confirmed influenza subtype or lineage.

The distribution of laboratory influenza admissions is described by type, subtype or lineage, site, and epidemiological week. Sociodemographic, clinical, vaccination, and severity parameters (death in hospital, ICU admission, and length of stay in hospital) are described for influenza test-positive and influenza test-negative virus, and by subtype of influenza virus. Parameters that are not normally distributed are log-transformed prior to analysis.

#### Heterogeneity

Heterogeneity is assessed using the *I*^2^ statistic.^24^ We explore potential sources of heterogeneity by conducting ad hoc subgroup analyses by study site, influenza virus type and subtype, and age group (<65 or ≥65 years). We define heterogeneity as low if *I*^2^ is <25%, moderate if *I*^2^ is 25–49%, and high if *I*^2^ is ≥50%, as described previously.^24^ We use forest plots to present the effect size of each risk factor for each study site and, in view of potential heterogeneity, all data are synthesized with random effects models.^17^ Analyses are restricted to subjects enrolled in the periods defined by the weeks in which positive specimens for influenza are ascertained at each site.^25^

#### Epidemiology and disease burden

Univariable analyses are used to assess whether risk factors for influenza in hospitalized cases are present in our study population. In the analysis estimating risk factors for a specific strain, we compare with test-negative for influenza patients exclusively or between strains.

Multivariable regression models are considered for these comparisons. Multivariable models including potential risk factors are used to calculate adjusted odds ratios (OR) of being influenza test-positive compared with influenza test-negative. We fit random effects logistic regressions models to account for the possible effect of data clustering by study site, including site as a cluster variable, and epidemiological week. Likelihood ratio tests are used to check whether the potential effects of clustering are significant.

Impact measures as the percentage of admissions attributable to influenza^26^ in the presence of a significant risk factor identified by the previous approaches are approximated by estimating average causal effects and potential outcomes by inverse probability weights logit analysis after taking into account confounders and exposure predictors.^27^

#### Influenza vaccine effectiveness

Vaccine effectiveness is estimated by the test-negative design^28^ as (1−[adjusted OR]) × 100.

Primary analyses estimate influenza vaccine effectiveness (IVE) among patients targeted for influenza vaccination according to site-specific vaccination policies and for those <65 years of age or ≥65 years of age.

Secondary outcome measures are hospital admissions with laboratory-confirmed influenza by subtype or lineage. In the analysis estimating vaccine effectiveness for a specific strain, those positive for other influenza strains are excluded.

The adjusted OR is estimated by logistic regression using a random effects model with study site as a shared parameter for the pooled analysis and including the confounders: week of symptom onset and age at admission, both modeled as restricted cubic splines. Also, the number of risk factors (including comorbidities, obesity, and pregnancy), smoking habits, previous access to healthcare, and time elapsed between illness onset and swabbing are included in the models of vaccine effectiveness as confounders or outcome predictors.

#### Sensitivity analysis

Sensitivity analyses using robust standard errors and generalized estimating equations are run. We also run a robust cluster-level analysis by working with the summary estimates in each cluster (study site) as the observations rather than the individual data.

In addition, for IVE estimates, sensitivity analyses are performed by including only samples taken within 4 days of symptom onset or considering subjects as vaccinated only if they had medical records of vaccine administration. A two-sided *P*-value < 0·05 is considered to indicate statistical significance.

#### Sample size

Assuming that 20% of admissions ascertained by applying the GIHSN protocol are related to influenza (Table S2), the number of influenza positives needed to estimate with a type 2 error <20% and a type 1 error ≤5%,^29^ overall or for a given risk group, the attributable risk of severe disease due to influenza infection as OR 1·25–1·50, is 1825 to 525 influenza-positive subjects, respectively. For instance, to detect an OR of two, the number of influenza positives needed would be 175.

**Figure 2:**
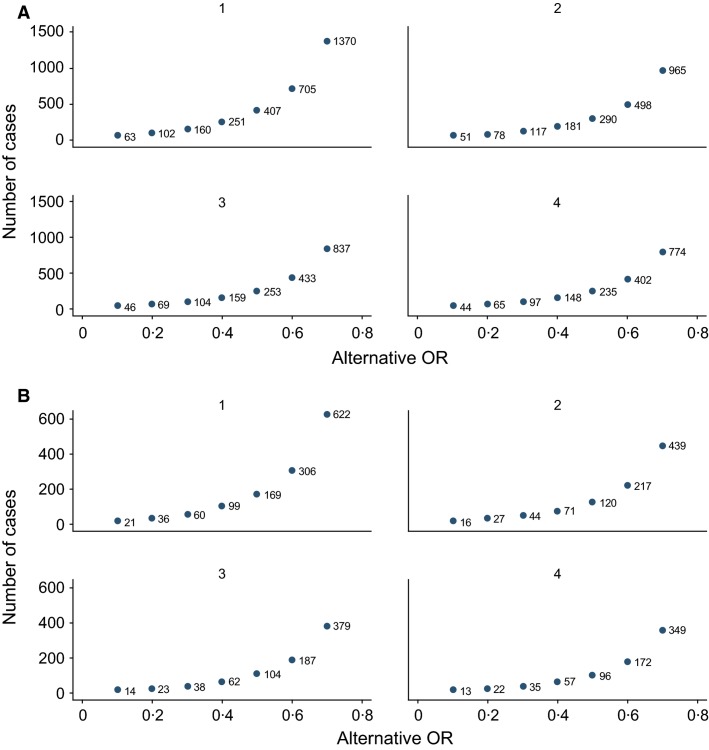
Number of cases to be included in any given subset (by group) analysis of vaccine effectiveness, depending on the number of matched controls per case. (A) Number of cases to be included when hospitalized controls have an influenza vaccine uptake of 15%. (B) Number of cases to be included when hospitalized controls have an influenza vaccine uptake of 50%.

Assuming hospitalized controls vaccinated in a range of 15–50%, aiming at being able to detect a vaccine effectiveness of ≥50% with a type 2 error of <20% and a type 1 error of ≤5%,^29^ the number of cases to be included in any given subset (overall, by age group, risk factor, or type or subtype analysis), depending on the number of controls per case, is shown in Figure2A and B. Figure2A gives the number of cases to be included in a scenario in which vaccine uptake is 15%, and Figure2B gives the same number when the expected coverage is 50%. In those two scenarios, the number of positive cases needed to detect a 50% vaccine effectiveness, allowing for adjustment for at least hospital and week, would be 290 or 130, respectively.

## Results

The GIHSN has run its activities for two consecutive influenza seasons, 2012–2013 and 2013–2014, and is well into its third season. Consistency on the application of the protocol and heterogeneity for the first season, 2012–2013, has been addressed in two previous publications.^5^,^17^

For the first season, the study was run in France, Russian Federation (Moscow and St. Petersburg), Turkey (as a pilot study), and Spain (Valencia). For the second season, the study was run in Brazil (three sites ran a pilot feasibility study, Fortaleza, Rio de Janeiro, and Porto Alegre), China [Beijing and Zhejiang (pilot study)], Russian Federation (Moscow and St. Petersburg), Turkey, and Valencia (Spain). For the third season, activities will be run in Brazil, Czech Republic, China, Russian Federation (Moscow and St. Petersburg), Turkey, and Spain (Valencia).

To show the feasibility and potential of the GIHSN active surveillance approach, we present the overall results of the GIHSN thus far. During the first two seasons, 19 677 eligible admissions were recorded; 11 843 (60%) were included and 2713 (23%) were positive for influenza. The major characteristics of the patients included are shown in Table S2. Overall, influenza-negative patients had more comorbidities, had more previous recent contacts with their physicians, had been hospitalized more times in the previous 12 months, had received influenza vaccination more often, and were more likely to die in hospital compared with influenza-positive admissions. In contrast, 60% of discharge diagnoses in influenza positives were due to pneumonia and influenza, compared with 18% in influenza-negative patients.

**Figure 3:**
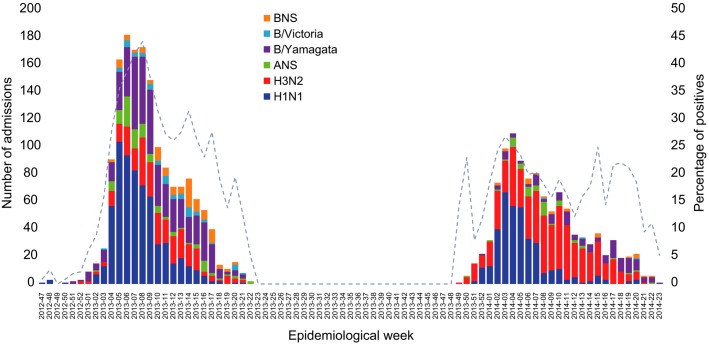
Influenza admissions by epidemiological week and season. ANS, influenza A not subtyped; BNS, influenza B not subtyped.

Influenza A predominated (Figure3) in the two seasons surveyed. Among influenza positives, we identified 991 (37%) A(H1N1); 807 (30%) A(H3N2); 583 (21%) B/Yamagata; 56 (2%) B/Victoria and 151 (6%) influenza A; and 125 (5%) influenza B that were not further characterized (Figure3 and Table1).

**Table 1:** Characteristics of influenza-positive admissions by type and subtype (the GIHSN first two seasons)

Characteristics	H1N1	H3N2	A not subtyped	B/Yamagata	B/Victoria	B not subtyped	Total
	991	807	151	583	56	125	2713
	%	%	%	%	%	%	
Age year, median (IQR)	31·3 (11·6–59·7)	25·5 (3·9–52·8)	17·0 (1·6–42·5)	34·4 (5·1–68·2)	24·6 (12·0–27·9)	24·7 (7·0–35·2)	26·0 (2·2–65·5)
Age group							
0–<5 years	22·4	27·6	44·4	24·0	23·2	20·0	25·4
5–<18 years	3·8	8·7	6·6	12·7	3·6	15·2	7·9
18–<50 years	39·2	37·6	29·8	24·2	66·1	50·4	36·0
50–<65 years	15·5	6·8	10·6	11·5	1·8	7·2	11·1
≥65 years	19·1	19·3	8·6	27·6	5·4	7·2	19·6
Sex							
Male	46·3	47·5	55·6	48·4	53·6	46·4	47·8
Female	53·7	52·5	44·4	51·6	46·4	53·6	52·2
Heart disease	15·4	17·2	6·6	16·5	8·9	11·2	15·4
COPD	14·0	10·3	8·6	12·9	0·0	1·6	11·5
Asthma	5·5	4·3	1·3	3·8	1·8	2·4	4·3
Diabetes	10·7	6·7	4·0	9·6	1·8	0·0	8·2
Immunodeficiency	1·1	2·1	1·3	1·9	3·6	0·0	1·6
Chronic renal disease	5·3	4·3	1·3	5·0	1·8	0·8	4·4
Neuromuscular disease	1·5	2·4	0·7	1·9	1·8	0·8	1·8
Cirrhosis liver disease	2·5	1·2	2·0	1·4	3·6	2·4	1·9
Neoplasm	3·0	3·0	1·3	2·7	0·0	1·6	2·7
Pregnancy	17·6	17·6	4·0	7·2	25·0	28·8	15·3
Obesity							
Underweight (BMI < 18·5)	23·0	31·7	45·0	34·0	25·0	27·2	29·4
Normal (18·5 ≤ BMI < 25)	38·0	36·7	30·5	34·1	51·8	41·6	36·8
Overweight (25 ≤ BMI < 30)	23·0	19·2	14·6	18·7	19·6	20·8	20·3
Obese (30 ≤ BMI < 40)	13·2	10·8	8·0	11·3	3·6	8·8	11·4
Morbidly obese (BMI ≥ 40)	2·4	0·9	0·7	1·4	0·0	0·8	1·5
Smoking habits							
Current smoker	22·2	19·3	20·5	13·4	21·4	16·0	19·1
Past smoker	18·4	15·9	8·0	14·2	14·3	16·8	16·0
Never smoker	56·4	63·4	68·9	70·3	60·7	54·4	62·2
Occupational socioeconomic class							
Qualified	30·8	41·6	27·2	23·0	35·7	65·6	33·8
Skilled	10·9	15·0	13·9	9·3	12·5	12·0	12·0
Lower, unskilled	28·1	12·9	14·6	21·3	10·7	5·6	19·9
Unknown	29·4	27·9	44·4	44·3	41·1	15·2	32·6
GP consultations in the past 3 months							
None	48·8	38·9	54·3	39·1	57·1	60·0	44·8
One	23·2	19·1	18·5	22·1	14·3	16·0	21·0
Two or more	27·0	40·2	22·5	34·5	19·7	22·4	31·9
Hospitalized in the past 12 months	18·8	25·0	14·6	18·4	12·5	11·2	19·8
Vaccination							
Seasonal 2010/11 flu vaccine*	9·1	14·9	8·8	20·9	11·3	12·8	13·7
Seasonal 2011/12 flu vaccine	14·4	11·8	8·0	16·5	12·5	10·4	13·5
Seasonal 2012/13 flu vaccine	14·5	11·4	9·3	14·4	1·8	4·8	12·6
Seasonal 2013/14 flu vaccine**	23·4	8·8	6·1	3·9	0·0	4·4	12·8
ICU	2·8	2·5	0·7	0·9	1·8	0·8	2·1
Exitus	1·6	1·1	0·0	1·4	0·0	0·0	1·2
Main discharge diagnosis							
Heart disease	1·3	2·0	0·0	1·5	0·0	0·0	1·4
COPD	7·0	4·5	0·7	7·2	0·0	0·8	5·5
Respiratory disease (other)	14·6	24·4	15·9	12·7	14·3	43·2	18·5
Pneumonia and influenza	64·7	55·3	74·2	56·4	73·2	49·6	60·1
Other	9·3	7·3	2·0	11·7	3·6	6·4	8·6

BMI, body mass index; COPD, chronic obstructive pulmonary disease; GP, general practitioner; ICU, intensive care unit; IQR, interquartile range. See footnote ^b^ in Table S2 for information on missing data.

* Only for the season 2012–2013.

** Only for the season 2013–2014.

## Discussion

The GIHSN platform can provide annual data on the severe end of the influenza infection spectrum, as represented by hospitalized cases, for a wide range of populations. The results should be generally applicable because of the diversity of participating hospitals and healthcare settings. Nevertheless, the adaptation of case identification to the specific local setting, mainly driven by practical considerations, will increase the sensitivity of the results to geographic variation. This will increase, its geographic representativeness and sample size, which will improve the validity and accuracy of data on influenza effects and their variability. This is especially important for attaining the principal public health objectives of preventing morbidity and premature mortality in people at high risk for complications from influenza.

The GIHSN study is bolstered by the active surveillance methodology; common criteria for inclusion across sites; a highly specific outcome definition of severe influenza, with influenza infection confirmed by RT-PCR; and the consistent body of evidence generated across sites.^5^,^17^

Existing networks that study hospitalizations with influenza tend to cover limited geographic areas in one country or continent, are based on routine surveillance systems aimed to detect epidemiological signals or evaluate trends, or are retrospective and rely on clinician-ordered testing and disease-coding practices.^15^,^30^ The GIHSN collects information on severe disease by actively searching hospital admission logs for eligible cases, patients are tested according to the study protocol, and the GIHSN study is run over several consecutive seasons in locations around the world.

Characteristics between sites and inside sites have a clear impact on the interpretation of the results reported as they reflect the relationship between the local influenza epidemiology and patients admitted in different-profile participating hospitals. This heterogeneity across sites is an opportunity to learn from the similarities and differences of the epidemiological patterns and differences between influenza virus types and subtypes, and their potential interactions. The GIHSN approach is intended to cover limitations due to small numbers, lack of representativity, or doubts on selection and information bias.^8^

Another limitation is the difficulty in defining for every site robust population denominators and therefore producing estimations on the incidence of disease.^2^ In a multicentric and multicountry context, we will answer this limitation using relative impact measures that explain the burden of the disease in comprehensible terms.

Influenza vaccine effectiveness is estimated using the test-negative approach, which has been shown to give consistent results,^15^ and the analysis of IVE shall be restricted to periods with similar influenza circulation patterns.^17^,^25^

The results already published for the 2012–2013 season and the consistency of the patterns of influenza circulation observed in the network sites analogous to those described by the WHO^31^,^32^ for the consecutive seasons support the value of the contribution of this public–private partnership to the global understanding of the epidemiology of influenza-related severe outcomes.

The breadth and consistency of results provided by the GIHSN will be particularly useful for assessing the effectiveness and cost-effectiveness of intervention programs, and they open the door to further research and public health activities, especially on risk factors and the impact of influenza and influenza vaccination programs in different populations. Thus, the GIHSN, with its broad representativeness and sustainable framework, should continue to contribute significantly to our knowledge of influenza epidemiology.

Future challenges lie in providing continuous proof of the quality of the data collected, improving the depth and quality of the analysis, reporting and interpretation, and in providing all of this information in a timely manner and in a way that contributes to the public health mission to improve people's health globally.

## APPENDIX A

The GIHSN Group members: F Aktaş, Faculty of Medicine, Gazi University, Ankara, Turkey; S Badur, National Influenza Reference Laboratory Capa-Istanbul, Istanbul, Turkey; A Buigues-Vila, FISABIO-Salud Pública, Valencia, Spain; S Borekci, Cerrahpaşa Faculty of Medicine, Istanbul University, Istanbul, Turkey; E Burtseva, D.I. Ivanovsky Institute of Virology, Moscow, Russian Federation; B Çakir, Department of Public Health, Faculty of Medicine, Hacettepe University, Sıhhiye, Ankara, Turkey; M Carballido, Hospital General, Castellón, Spain; C Carratalá-Munuera, Universidad Miguel Hernández, San Juan de Alicante, Spain; E Chen, Zhejiang Provincial Center for Disease Control and Prevention, Hangzhou, China; MA Ciblak, National Influenza Reference Laboratory Capa-Istanbul, Istanbul, Turkey; S Çelebi, Uludağ University Faculty of Medicine, Bursa, Turkey; BJ Cowling, School of Public Health, Li Ka Shing Faculty of Medicine, The University of Hong Kong, China; DB Deniz, Dr. Siyami Ersek Göğüs Kalp ve Damar Cerrahisi Eğitim ve Araştırma Hastanesi Istanbul, Turkey; M Durusu, Hacettepe Üniversitesi, Ankara, Istanbul, Turkey; C El Guerche-Seblain, Sanofi Pasteur, Lyon, France; L Feng, Key Laboratory of Surveillance and Early-warning on Infectious Disease, Division of Infectious Disease, Chinese Center for Disease Control and Prevention, Beijing, China; S Gencer, Dr. Lütfi Kırdar Kartal Training and Research Hospital, Istanbul, Turkey; V Gil-Guillén, Universidad Miguel Hernández, San Juan de Alicante, Spain; M Hacımustafaoğlu, Uludağ University, Bursa, Turkey; S Hancerli, Istanbul Faculty of Medicine, Istanbul University, Istanbul, Turkey; DK Ip, School of Public Health, The University of Hong Kong, China; L Kisteneva, D.I. Ivanovsky Institute of Virology, Moscow, Russian Federation; L Kolobukhina, D.I. Ivanovsky Institute of Virology, Moscow, Russian Federation; R Limón-Ramírez, Hospital La Plana, Vila-real, Spain; FX López-Labrador, FISABIO-Salud Pública, Valencia, Spain, and Consorcio de Investigación Biomédica de Epidemiología y Salud Pública (CIBERESP), Instituto de Salud Carlos III, Madrid, Spain; C Mahé, Sanofi Pasteur, Lyon, France; A Mira Iglesias, FISABIO-Salud Pública, Valencia, Spain; J Mollar Maseres, Hospital La Fe, Valencia, Spain; A Natividad-Sancho, FISABIO-Salud Pública, Valencia, Spain; L Ozisik, Hacettepe University, Ankara, Turkey; L Osidak, Research Institute of Influenza, St. Petersburg, Russian Federation; MC Otero-Reigada, Hospital La Fe, Valencia, Spain; S Özer, Dr. Lütfi Kırdar Kartal Training and Research Hospital, Istanbul, Turkey; M Pisareva, Research Institute of Influenza, St. Petersburg, Russian Federation; J Puig-Barberà, FISABIO, Valencia, Spain; Y Qin, Key Laboratory of Surveillance and Early-warning on Infectious Disease, Division of Infectious Disease, Chinese Center for Disease Control and Prevention, Beijing, China; A Eren-Şensoy, Dr. Siyami Ersek Göğüs Kalp ve Damar Cerrahisi Eğitim ve Araştırma Hastanesi, Istanbul, Turkey; A Sominina, Research Institute of Influenza, St. Petersburg, Russian Federation; K Stolyarov, Research Institute of Influenza, St. Petersburg, Russian Federation; A Tormos, FISABIO, Valencia, Spain; M Tortajada-Girbés, Hospital Dr Peset, Valencia, Spain; S Trushakova, D.I. Ivanovsky Institute of Virology, Moscow, Russian Federation; Q Wang, Beijing Center for Disease Prevention and Control, Beijing, China; P Wu, School of Public Health, The University of Hong Kong, China; H Yu, Key Laboratory of Surveillance and Early-warning on Infectious Disease, Division of Infectious Disease, Chinese Center for Disease Control and Prevention, Beijing, China; J Zheng, Key Laboratory of Surveillance and Early-warning on Infectious Disease, Division of Infectious Disease, Chinese Center for Disease Control and Prevention, Beijing, China.

## Supporting Information

Table S1 Clinical conditions used to identify admissions possibly associated with an influenza infection. **Table S2** Characteristics of influenza-positive and influenza-negative patients.
