# Supplementary material for: The Global Influenza Hospital Surveillance Network (GIHSN): a new platform to describe the epidemiology of severe influenza
Source: Influenza Other Respir Viruses. 2015 Oct 13;9(6):277–86. doi: 10.1111/irv.12335 (PMC4605407; doi:10.1111/irv.12335)
Supplement: Table S1 — Clinical conditions used to identify admissions possibly associated with an influenza infection. Table S2 Characteristics of influenza-positive and influenza-negative patients. [file irv0009-0277-sd1.docx]

**Supporting Table S1.** Clinical conditions used to identify admissions possibly associated with an influenza infection

| **Patients ≥5 years of age** | **ICD 9 Codes** | **ICD 10 Codes** |
| --- | --- | --- |
| Acute respiratory infection | 382·9; 460–466 | J00–J06, J20–J22, H66·90 |
| Acute myocardial infarction or acute coronary syndrome | 410–411 and 413–414 | I20–I25·9 |
| Asthma | 493–493·92 | J45·2–J45·22, J45·9–J45·998, J44–J44·9 |
| Heart failure | 428–429·0 | I50–I50·9; I51·4 |
| Pneumonia and influenza | 480–488 | J09–J18 |
| Chronic pulmonary obstructive disease | 490, 491, 492, 496 | J40–J44·9 |
| Myalgia | 729·1 | M79·1 |
| Metabolic failure (diabetic coma, renal dysfunction, acid-base disturbances, alterations to the water balance) | 250·1– 250·3; 584–586; 276–277 | E11·9, E10·9, E11·65, E10·65, E10·11, E11·01, E10·641, E11·641, E10·69, E11·00, E10·10, E11·69, N17·0, N17·1, N17·2, N17·8, N17·9, N18·1, N18·2, N18·3, N18·4, N18·5, N18·6M N18·9, N19, E87·0, E87·1, E87·2, E87·3, E87·4, E87·5, E87·6, E87·70, E87·71, E87·79, E86·0, E86·1 |
| Altered consciousness, convulsions, febrile-convulsions | 780·01–780·02; 780·09; 780·31–780·32 | R40·20, R40·4, R40·0, R40·1, R56·00, R56·01 |
| Dyspnea/respiratory abnormality | 786·0 | R06·0, R06–R06·9 |
| Respiratory abnormality | 786·00 | R06·9 |
| Shortness of breath | 786·05 | R06·02 |
| Respiratory abnormality NEC | 786·09 | R06·3, R06·00, R06·09, R06·83 |
| Respiratory symptoms/chest symptoms | 786·9 | R06·89 |
| Fever or fever unknown origin or non-specified | 780·6–780·60 | R50, R50·9 |
| Cough | 786·2 | R05 |
| Sepsis, systemic inflammatory response syndrome | 995·90–995·94 | R65·10, R65·11, R65·20, A41·9 |
| **Patients 0–4 years of age** | **ICD 9 Codes** | **ICD 10 Codes** |
| Acute upper or lower respiratory disease | 382·9; 460 to 466 | J00–J06, J20–J22 |
| Dyspnea, breathing anomaly, shortness of breath, tachypnea | 786·0; 786·00; 786·05–786·07; 786·09; 786·9 | R06·0, R06, R06·9, R06·3, R06·00, R06·09, R06·83, R06·02, R06·82, R06·2, R06·89 |
| Asthma | 493–493·92 | J45·2–J45·22, J45·9–J45·998, J44–J44·9 |
| Pneumonia and influenza | 480 to 488 | J09–J18 |
| Heart failure | 428–429·0 | I50–I50·9; I51·4 |
| Myalgia | 729·1 | M79·1 |
| Altered consciousness, convulsions, febrile convulsions | 780·01–780·02; 780·09; 780·31–780·32 | R40·20, R40·4, R40·0, R40·1, R56·00, R56·01 |
| Fever or fever unknown origin or non-specified | 780·6–780·60 | R50, R50·9 |
| Cough | 786·2 | R05 |
| Gastrointestinal manifestations | 009·0; 009·3 | A09·0; A09·9 |
| Sepsis, systemic inflammatory response syndrome | 995·90–995·94 | R65·10, R65·11, R65·20, A41·9 |

**Supporting Table S2.** Characteristics of influenza-positive and influenza-negative patients^a,b^ (GIHSN, 2012–2013 and 2013–2014 influenza seasons)

|  | **Influenza negatives** | **Influenza positives** | **Total** | ***P*-value** |
| --- | --- | --- | --- | --- |
|  | **9130** | **2713** | **11 843** |  |
| **Characteristic** | **%** | **%** | **%** |  |
| **Age y, median (IQR)** | 24·9 (1·8–68·0) | 27·9 (1·2–57·1) | 25·6 (2·2–65·5) | 0·0002 |
| **Age group** |  |  |  | < 0·0001 |
| 0–<5 y | 3445 (37·7) | 690 (25·4) | 4135 (34·9) |  |
| 5–<18 y | 493 (5·4) | 213 (7·9) | 706 (6·0) |  |
| 18–<50 y | 1936 (21·2) | 977 (36·0) | 2913 (24·6) |  |
| 50–<65 y | 777 (8·5) | 302 (11·1) | 1079 (9·1) |  |
| ≥65 y | 2478 (27·1) | 531 (19·6) | 3009 (25·4) |  |
| **Sex** |  |  |  | < 0·0001 |
| Male | 5132 (56·2) | 1296 (47·8) | 6428 (54·3) |  |
| Female | 3997 (43·8) | 1417 (52·2) | 5414 (45·7) |  |
| **Heart disease** | 1618 (17·7) | 417 (15·4) | 2035 (17·2) | 0·0004 |
| **COPD** | 1579 (17·3) | 312 (11·5) | 1891 (16·0) | < 0·0001 |
| **Asthma** | 413 (4·5) | 117 (4·3) | 530 (4·5) | 0·8370 |
| **Diabetes** | 987 (10·8) | 223 (8·2) | 1210 (10·2) | < 0·0001 |
| **Immunodeficiency** | 109 (1·2) | 43 (1·6) | 152 (1·3) | 0·1120 |
| **Chronic renal disease** | 432 (4·7) | 120 (4·4) | 552 (4·7) | 0·5020 |
| **Neuromuscular disease** | 205 (2·3) | 48 (1·8) | 253 (2·1) | 0·1320 |
| **Cirrhosis liver disease** | 165 (1·8) | 51 (1·9) | 216 (1·8) | 0·8050 |
| **Neoplasm** | 316 (3·5) | 74 (2·7) | 390 (3·3) | 0·0600 |
| **Pregnancy** | 468 (5·1) | 414 (15·3) | 882 (7·5) | < 0·0001 |
| **Obesity** |  |  |  | < 0·0001 |
| Underweight (BMI<18·5) | 3241 (35·5) | 798 (29·4) | 4039 (34·1) |  |
| Normal (18·5≤BMI<25) | 2881 (31·6) | 999 (36·8) | 3880 (32·8) |  |
| Overweight (25≤BMI<30) | 1813 (19·9) | 551 (20·3) | 2364 (20·0) |  |
| Obese (30≤BMI<40) | 1016 (11·1) | 309 (11·4) | 1325 (11·2) |  |
| Morbidly obese (BMI≥40) | 122 (1·3) | 41 (1·5) | 163 (1·4) |  |
| **Smoking habits** |  |  |  | < 0·0001 |
| Current smoker | 1743 (19·1) | 517 (19·1) | 2260 (19·1) |  |
| Past smoker | 1788 (19·6) | 434 (16·0) | 2222 (18·8) |  |
| Never smoker | 5338 (58·5) | 1687 (62·2) | 7025 (59·3) |  |
| **Occupational socioeconomic class** |  |  |  | < 0·0001 |
| Qualified | 2999 (32·9) | 918 (33·8) | 3917 (33·1) |  |
| Skilled | 1173 (12·9) | 326 (12·0) | 1499 (12·7) |  |
| Lower-unskilled | 2923 (32·0) | 541 (19·9) | 3464 (29·3) |  |
| Unknown | 1846 (20·2) | 883 (32·6) | 2729 (23·0) |  |
| **GP consultations in the past 3 months** |  |  |  | < 0·0001 |
| None | 3017 (33·0) | 1215 (44·8) | 4232 (35·7) |  |
| One | 2033 (22·3) | 569 (21·0) | 2602 (22·0) |  |
| Two or more | 3907 (42·8) | 865 (31·9) | 4772 (40·3) |  |
| **Has been hospitalized in the past 12 months?** | 2207 (24·2) | 538 (19·8) | 2745 (23·2) | < 0·0001 |
| **Vaccination** |  |  |  |  |
| Seasonal 2010–2011 flu vaccine^c^ | 1061 (23·8) | 219 (13·7) | 1280 (21·1) | < 0·0001 |
| Seasonal 2011–2012 flu vaccine | 1897 (20·8) | 366 (13·5) | 2263 (19·1) | < 0·0001 |
| Seasonal 2012–2013 flu vaccine | 1951 (21·4) | 341 (12·6) | 2292 (19·4) | < 0·0001 |
| Seasonal 2013–2014 flu vaccine^d^ | 806 (17·2) | 142 (12·8) | 948 (16·4) | < 0·0001 |
| **ICU** | 176 (1·9) | 56 (2·1) | 232 (2·0) | 0·5360 |
| **Exitus** | 176 (1·9) | 33 (1·2) | 209 (1·8) | 0·0130 |
| **Main discharge diagnosis** |  |  |  |  |
| Heart disease | 313 (3·4) | 38 (1·4) | 351 (3·0) | < 0·0001 |
| COPD | 674 (7·4) | 149 (5·5) | 823 (7·0) | 0·5800 |
| Respiratory disease (other) | 4396 (48·1) | 502 (18·5) | 4898 (41·4) | < 0·0001 |
| Pneumonia and influenza | 1658 (18·2) | 1631 (60·1) | 3289 (27·8) | < 0·0001 |
| Other | 1710 (18.7) | 232 (8·6) | 1942 (16·4) | < 0·0001 |

BMI, body mass index; COPD, chronic obstructive pulmonary disease; GP, general practitioner; ICU, intensive care unit; IQR, interquartile range.

^a^Admissions in consenting non-institutionalized permanent residents admitted because of complaints possibly related to influenza that began within 7 days of admission for children <5 years of age, or that, for patients ≥5 years, in addition to the complaints possibly related to influenza, had an influenza-like illness within 7 days of admission were included in the study. Included subjects were systematically swabbed without previous knowledge of influenza or vaccination for influenza status.

^b^All percentages are estimated including missing data. Data on age and sex was missing in one subject, in 10 subjects (<0.1%) there was no data on chronic conditions, for chronic asthma data was missing in 456 (3.9%), 447 subjects with missing data on the presence or not of chronic asthma belonged to one site that contributed with 447 subjects and participated in the network only during the first season. Data on weight or height was missing in 72 (0.6%) subjects, smoking habits in 336 (3%) social class was missing in 234 (2%), information on frequency of outpatient consultations and hospital admissions was missing in 237 (2%) and 263 (2%), respectively, intensive care unit admission was missing in 462(4%), again, this information was missing in the 447 subjects from the previous mentioned site, death in hospital in 56 (0.5%) and main discharge diagnoses in 536 (5%), 429 of those were subjects from the mentioned site. Information on current season vaccination was complete, and was only missing in 14 and 18 cases (<0.4%) for the previous season (2012/13 and 2013/14) influenza vaccine.

^c^Only for the season 2012–2013.

^d^Only for the
